# Long Non-Coding RNAs: Critical Players in Hepatocellular Carcinoma

**DOI:** 10.3390/ijms151120434

**Published:** 2014-11-07

**Authors:** Jin Sun, Beibei Bie, Shu Zhang, Jun Yang, Zongfang Li

**Affiliations:** 1 National-Local Joint Engineering Research Center of Biodiagnostics and Biotherapy, Xi’an Jiaotong University, Xi’an 710004, China; E-Mails: jinsun2012@outlook.com (J.S.); biepeipei@mail.xjtu.edu.cn (B.B.); drzhangshu@163.com (S.Z.); yangjundr@163.com (J.Y.); 2 Department of General Surgery, the Second Affiliated Hospital, School of Medicine, Xi’an Jiaotong University, Xi’an 710004, China; 3 Department of Pathology, the Second Affiliated Hospital, School of Medicine, Xi’an Jiaotong University, Xi’an 710004, China

**Keywords:** long noncoding RNA, hepatocellular carcinoma, dysregulation, biological roles, molecular mechanism

## Abstract

Hepatocellular carcinoma (HCC) is a complex disease with multiple underlying pathogenic mechanisms caused by a variety of etiologic factors. Emerging evidence showed that long non-coding RNAs (lncRNAs), with size larger than 200 nucleotides (nt), play important roles in various types of cancer development and progression. In recent years, some dysregulated lncRNAs in HCC have been revealed and roles for several of them in HCC have been characterized. All these findings point to the potential of lncRNAs as prospective novel therapeutic targets in HCC. In this review, we summarize known dysregulated lncRNAs in HCC, and review potential biological roles and underlying molecular mechanisms of lncRNAs in HCC. Additionally, we discussed prospects of lncRNAs as potential biomarker and therapeutic target for HCC. In conclusion, this paper will help us gain better understanding of molecular mechanisms by which lncRNAs perform their function in HCC and also provide general strategies and directions for future research.

## 1. Introduction

Hepatocellular carcinoma (HCC) is one of the most common cancers in the world as more than 700,000 cases are diagnosed and approximately 600,000 deaths are reported annually, especially in, East and Southeast Asia, Africa and Southern Europe [1,2]. This disease is often associated with an extremely poor prognosis because patients are either diagnosed at a very late stage or experience recurrence and metastasis after surgical resection [3]. It is well known that a variety of risk factors have been associated with the incidence of HCC, such as hepatitis B virus (HBV) and hepatitis C virus (HCV) infection, aflatoxin B1 intake, tobacco smoking, alcoholic cirrhosis, and so on [4]. Poor understanding of the mechanisms underlying the pathogenesis of HCC makes it difficult to be diagnosed and treated at an early stage, thus an urgent needs to elucidate the molecular mechanisms underlying HCC and to identify and provide effective targets for therapy or early detection of HCC. Although significant advances have been made in recent decades, our understanding of the underlying molecular mechanisms of HCC remains limited and these investigations have largely focused on the role of protein-coding genes and some classic epigenetic factors, including microRNAs (miRNAs), DNA methylation, and several types of histone modifications involving histone methylation and acetylation [4,5,6,7,8,9,10].

In recent years, advancements in genome-wide analyses of the mammalian transcriptome have revealed a novel class of transcripts, long noncoding RNAs (lncRNAs), which are pervasively transcribed in the genome [11]. LncRNAs are arbitrarily defined as transcripts of more than 200 nucleotides (nt) in length that lack significant open reading frames (ORF) and can be localized to both the nucleus and cytoplasm [12,13]. Accumulating evidence indicates that lncRNAs are not the “dark matter” of the genome, but that they play significant roles in various biological processes through complicated mechanisms, including X-inactivation, genomic imprinting, cell differentiation, cell apoptosis, stem cell pluripotency, nuclear trafficking, heat shock response, and genome rearrangement [14]. It is noteworthy that an increasing number of studies have demonstrated lncRNAs as a new class of regulatory molecules that are involved in a variety of human disease, especially cancer, through modulating gene expression at transcriptional, post-transcriptional or epigenetic level [15]. Some classical lncRNAs have been found to be dysregulated in a variety of cancers and have been shown to possess clinical potential as diagnostic biomarkers and therapeutic targets due to their aberrant expression is significantly associated with carcinogenesis, metastasis or prognosis, such as *H19*, *HOTAIR*, *MALAT1*, *MEG3*, and *XIST* [16,17]. HCC, as the most common type of primary liver cancer, is revealing the potential roles of lncRNAs in hepatocellular carcinoma and is attracting increased attention in cancer research in recent years. Excitingly, some significant and substantial progress has been made toward identifying and functional characterizing HCC-related lncRNAs.

In this review, we focus our attention on lncRNAs that are involved in HCC. Firstly, we summarize known dysregulated lncRNAs in HCC, and then we review potential biological roles and underlying molecular mechanisms of lncRNAs in HCC. Finally, we discuss prospects of lncRNAs as potential biomarker and therapeutic target for HCC.

All in all, this paper will help us gain a better understanding of molecular mechanisms by which lncRNAs perform their function in HCC and also provide general strategies and directions for future research.

## 2. Dysregulation of Long Non-Coding RNAs (lncRNAs) in Hepatocellular Carcinoma

It has been shown that most lncRNAs are expressed in a tissue/cell type-specific pattern or in a developmental stage specific manner and are transcribed by RNA polymerase II (RNA Pol II), as well as possess a 5'-methyl cap and a polyadenylated tail, similar to mRNAs, indicating these lncRNAs can be tightly regulated and may play specific biological roles in a variety of biological processes and human disease, and can also lead to undesired biological consequences when dysregulated. Indeed, a growing body of evidence has indicated that dysfunctional lncRNAs are implicated in a broad range of cancers, including HCC. So far, a handful of dysregulatd lncRNAs that are associated with HCC have been identified (see Table 1).

*H19*, a 2.3-kb lncRNA, is a famous paternally imprinted (maternally expressed) gene and is highly expressed from the early stages of embryogenesis to fetal life in many organs but is almost entirely down-regulated postnatally and it plays important roles in embryonic development and growth control [18]. Emerging evidence showed that erasure of *H19* imprinting and subsequent high expression level of *H19* was associated with tumor growth, metastasis and invasion of several types of cancer. [19,20,21]. Kim and Lee (1997) firstly found that the expression of *H19* usually shift from monoallelic to biallelic in HCC and it might play a causal role in the epigenetic mechanism involved in tumor development and/or process [22]. Later, *H19* RNA level was shown to be up-regulated in HBV-associated HCC [23]. Additionally, Matouk *et al*. (2007 and 2010) revealed that hypoxia could strongly up-regulated the level of *H19* RNA in the HCC cell line [24,25].

*HOTAIR* (*Homeobox* antisense intergenic RNA), an ncRNA with a length of 2158 bp, is transcribed from the antisense strand of *homeobox C* gene locus in chromosome 12. A large number of studies have shown that *HOTAIR* is up-regulated in various cancers and correlates with carcinogenesis and metastasis, as well as poor prognosis [26]. A previous study from Geng *et al*. reported that *HOTAIR* expression was significantly higher in hepatocellular carcinoma (HCC) tissue than that in adjacent noncancerous tissues [27]. A recent study performed in HCC also found that *HOTAIR* was overexpressed in HCC patients and was associated with a worse prognosis and an increased risk of metastasis in these patients [28].

*HOTTIP* (*HOXA* transcript at the distal tip), an lncRNA transcribed from the 5' end of the *HOXA* locus that regulates the activation of some *HOXA* genes *in vivo* [29]*.* A recent study from Quagliata *et al*. reported that *HOTTIP* was significantly up-regulated in HCC specimens and its high expression level was associated with metastasis formation and poor patient survival in HCC [30].

*HULC* (highly up-regulated in liver cancer), a 500 bp spliced lncRNA, was first identified as a novel mRNA-like noncoding RNA that up-regulated remarkably in HCC by Panzitt *et al*. [31]. *HULC* expression has been reported to be regulated by the transcription factor CREB (cyclic adenosine monophosphate responsive element binding protein) in Hep3B cells [32]. Interestingly, the expression level of *HULC* is positively associated with those of hepatitis B virus X protein (HBx) in clinical HCC tissues. Moreover, HBx could up-regulate *HULC* expression level in L-O2 cells (a human immortalized normal liver cell line) and HepG2 cells (a human hepatoma cell line) [33].

*KCNQ1OT1* (potassium voltage-gated channel, KQT-like subfamily, member 1 overlapping transcript 1), a maternally imprinted lncRNA transcribed from *KCNQ1* locus and responsible for transcriptional silencing a bunch of genes at *KCNQ1* locus *in cis* by modulating histone methylation, has been found to be involved in various types of cancers [34]. A recent study showed that a short tandem repeat (STR) polymorphism (rs35622507) within the *KCNQ1OT1* coding region was identified as the risk conferring polymorphism for HCC in the Chinese population and a significant genotype–phenotype correlation in which the protective genotypes (heterozygote and non-10) of the STR polymorphism confer increased *KCNQ1OT1* expression and partially decreased *CDKN1C* expression *in vitro* [34].

*Linc-RoR* (long non-coding RNA regulator of reprogramming), a large intergenic noncoding RNA with a length of 2.6-kb, was previously identified as a key reprogramming regulator and whose expression is connected to pluripotency via regulating the key pluripotency transcription factors (TFs) including Oct4, Sox2, and Nanog as a competing endogenous RNA (ceRNA) [35,36]. Interestingly, Takahashi *et al*. reveled that expression of *linc-RoR* was up-regulated in malignant cells compared to non-malignant hepatocytes and increased in responses to hypoxia [37].

*MALAT1* (metastasis-associated lung adenocarcinoma transcript1), an lncRNA originally identiﬁed to be overexpressed in patients at high risk for metastasis of non-small cell lung tumors (NSCLC), was up-regulated in many solid tumors and associated with cancer metastasis and recurrence [38]. *MALAT1* has been shown to be up-regulated in HCC cell lines and clinical tissue samples [38,39].

*MEG3* is a maternal imprinted gene highly expressed in the human pituitary. It is able to interact with cyclic AMP, p53 (Tumor protein p53), and growth differentiation factor 15 (GDF15) and plays an important role in cell proliferation control. Decrease of *MEG3* expression has been observed in several types of cancer [40]. Huang *et al*. (2007) found that *MEG3* is down-regulated in HCC compared to normal liver tissues [41]. A later study also showed that *MEG3* expression was markedly reduced in four human HCC cell lines compared with normal hepatocytes, and overexpression of *MEG3* in HCC cells dramatically inhibited HCC cell growth, as well as *MEG3* expression could be regulated by microRNA-29 [42].

*PCNA-AS1* (proliferating cell nuclear antigen antisense RNA 1), an antisense long noncoding RNAs located on the opposite strand of gene proliferating cell nuclear antigen (PCNA), was recently found to be significantly up-regulated in HCC compared with peritumoral tissues by Yuan *et al*. [43].

In particular, the unprecedented advances in high-throughput screening technologies, such as microarrays and transcriptome sequencing, facilitate large-scale identification and characterization of novel disease-related genes, including lncRNAs. Excitingly, some papers have revealed the lncRNA expression profiles in HCC samples and paired non-tumor samples using microarray, and a set of HCC-related lncRNAs have been identified. For example, *lncRNA-DREH* (down-regulated expression by HBx), an lncRNA differentially expressed between livers of HBx transgenic mice and wild-type mice, was identified by microarray and the expression level of its human ortholog RNA, *hDREH*, was frequently down-regulated in HBV-related HCC tissues in comparison with the adjacent noncancerous hepatic tissues, and its decrease significantly, dramatically, associated with poor survival in HCC patients [44]. By comparing the lncRNA expression profiles of HBV-related HCC and paired peritumoral tissue, Yang *et al*. found *lncRNA-HEIH* (high expression in HCC), one of differentially expressed lncRNA, was highly expressed in HBV-related HCC and was significantly correlated with recurrence [45]. *LncRNA-MVIH* (microvascular invasion in HCC), an lncRNA derived from microarray data that used for identification of *lncRNA-HEIH*, was also shown to be up-regulated in HCC [46]. *LncRNA-LET* (low expression in tumor), an lncRNA also derived from the same microarray data that used for identification of *lncRNA-HEIH*, was shown to be down-regulated in HCC [47]. By comparing lncRNA expression levels between TGF-β treated and untreated SMMC-7721 hepatoma cells using microarray, Yuan *et al*. found *lncRNA-ATB* (lncRNA activated by TGF-β), was highly expressed in HCC and associated with poor prognosis in HCC [48]. Additionally, a recent study revealed *lncRNA-hPVT1* (human plasmacytoma variant translocation 1), the human ortholog of *lncRNA-mPVT1* (mouse plasmacytoma variant translocation 1) that was a fetal liver-specific lncRNAs identified by microarray analysis, is significantly up-regulated in HCC tissues and high *hPVT1* expression is associated with poor prognosis in HCC patients [49].

*uc002mbe.2*, a TSA (Trichostatin A)-induced lncRNA, was strongly expressed in TSA-treated Huh7 cells. Yang *et al*. found that *uc002mbe.2* had more than 300-folds induction upon TSA treatment and its expression level was significantly lower in the HCC cell lines and liver cancer tissue compared with normal human hepatocytes and adjacent noncancerous tissues [50].

*URHC* (up-regulated in hepatocellular carcinoma), an lncRNA was highly expressed in hepatoma cells and HCC tissues and was originally identified by comparing lncRNA expression profiling of three HCC cell lines and normal hepatocytes using lncRNA microarray. Xu *et al*. revealed that the higher expression of *URHC* was correlated with poor overall survival [51].

**Table 1:** Dysregulatd long non-coding RNAs (lncRNAs) that are associated with HCC.

LncRNA	Dysregulation	Biological Functions in HCC	Molecular Mechanism	Reference
*H19*	Up-regulated	Promote HCC growth	Competitively bind to the *let-7*; interact with EZH2 and repress the expression of E-cadherin	[18,19,20,21,22,23,24,25]
*HOTAIR*	Up-regulated	Promote HCC growth	Competitively bind to the *miR-331-3p* and depress the expression of *HER2*; interact with EZH2 and LSD1	[27,28,52,53]
*HOTTIP*	Up-regulated	Promote proliferation of HCC cells	Interact with WDR5/MLL and drive the H3K4me3	[29,30]
*HULC*	Up-regulated	Promote HCC growth	Competitively bind to the *miR-372*	[31,32,33]
*KCNQ1OT1*	Up-regulated	Promote HCC progression	Interact with Ehmt2 and PRC2 complex and drive the H3K9 and H3K27 methylation	[34,54]
*Linc-ROR*	Up-regulated	Promote cell survival during hypoxic stress	Competitively bind to the *miR-145* Competitively bind to	[37]
*MALAT1*	Up-regulated	Promote invasion	miR-9 targets MALAT1 in the nucleus and regulates the MALAT1 in an AGO2-dependent manner; competitively bind to *miR-125b*	[38,39,55,56]
*MEG3*	Down-regulated	HCC growth control	Interact with PRC2 complex	[41,42,57]
*PCNA-AS1*	Up-regulated	Promote HCC growth	Form RNA hybridization to increase PCNA mRNA stability	[43]
*LncRNA-DREH*	Down-regulated	Inhibit growth and metastasis	Interact with vimentin protein and repress its expression	[44]
*LncRNA-HEIH*	Up-regulated	Promote HCC growth	Interact with EZH2 and repress the expression of *p15*, *p16*, *p21*, *p57*	[45]
*LncRNA-MVIH*	Up-regulated	Promote HCC growth, microvascular invasion and intrahepatic metastasis	Interact with PGK1 and inhibit its secretion	[46]
*LncRNA-LET*	Down-regulated	Inhibit hypoxia-induced HCC cell invasion	Interact with NF90 protein and increases its degradation	[47]
*LncRNA-ATB*	Up-regulated	Induced by TGF-β and promotes EMT, HCC cell invasion and metastasis	Competitively bind to the *miR-200* family; binding *IL-11* mRNA	[48]
*LncRNA-hPVT1*	Up-regulated	Promotes HCC growth	Interact with NOP2 protein and enhance its stability	[49]

Abbreviations: HER2, human epidermal growth factor receptor 2; Ehmt2, histone-lysine *N*-methyltransferase EHMT2; EZH2, enhancer of zeste homolog 2; ERK, extracellular signal-regulated kinases; IL-11, Interleukin 11; LDS1, lysine-specific demethylase 1; MAPK, mitogen-activated protein kinase; MLL, mixed lineage leukemia; PGK1, phosphoglycerate kinase 1; PRC2, polycomb repressive complex 2; WDR5, WD repeat domain 5; ZAK, mitogen-activated protein kinase kinase kinase MLT.

## 3. Biological Roles of lncRNAs in Hepatocellular Carcinoma

### 3.1. Hepatocellular carcinoma (HCC) Growth

To date, many lncRNAs dysregulated in HCC have been demonstrated to play important roles in HCC growth *in vitro* or *in vivo*. In the study conducted by Matouk *et al*., the authors found that ablations of tumorigenicity of HCC *in vivo* was seen by *H19* knockdown which also significantly abrogated anchorage-independent growth after hypoxia recovery [24]. *In vitro* assays in the HCC cell line Bel7402 demonstrated that knockdown of *HOTAIR* lincRNA could reduce cell proliferation [27]. Du *et al*. demonstrated that *HULC* could promote cell proliferation by MTT, colony formation assay, and tumorigenicity assay [33]. Quagliata *et al*. demonstrated that knockdown of *HOTTIP* could signiﬁcantly reduce cell proliferation of HuH-6 and HuH-7 cell lines [30]. Yang *et al*. revealed that knockdown of *lncRNA-HEIH* could inhibit the proliferation of HCC cell by affecting cell cycle and the growth of tumors from *lncRNA-HEIH*-down-regulated xenografts were significantly inhibited when compared with that of tumors formed from control xenografts [45]. Yang *et al*. also demonstrated that *lncRNA-MVIH* could promote HCC growth both *in vitro* and *in vivo* [46]. Huang *et al*. revealed that suppression of cellular *lncRNA-DREH* could enhance the cell proliferation effect *in vitro* and its overexpression could repress the growth of tumor *in vivo* [44]. Recently, Takahashi *et al*. uncovered that knockdown of *linc-RoR*, a hypoxia-responsive lncRNA, could decrease cell viability in HCC cells during hypoxia [37]. Yuan *et al*. demonstrated lncRNA *PCNA-AS1* could dramatically promote tumor growth *in vitro* and *in vivo* [43]. A recent study revealed *lncRNA-hPVT1* could promote cell proliferation, cell cycling and stem cell-like phenotype of HCC cells *in vitro* and promote HCC growth *in vivo* [49]. Additionally, another recent study demonstrated that *URHC* inhibition could reduce the proliferation of HCC cells [51].

### 3.2. HCC Invasion and Metastasis

It is well known that the poor prognosis and high recurrence rate of HCC is largely due to the high incidence of intrahepatic and extrahepatic metastases [58]. Thus, the inhibition of invasion and metastasis is of great importance in HCC therapies. There is now increasing evidence that lncRNAs play important roles in invasion and metastasis of HCC. For example, Huang *et al*. demonstrated that overexpression of *lncRNA-Dreh* could inhibit tumor metastasis *in vivo* by establishing orthotopic liver implanted metastatic models and peripheral intravascular implanted metastatic models [44]. Yuan *et al*. revealed that *lncRNA-MVIH* overexpression resulted in significantly frequent intrahepatic metastasis by establishing the liver metastasis tumor model [46]. Lai *et al*. found that inhibition of *MALAT1* in HepG2 cells could effectively reduce cell motility and invasiveness [39]. Yang *et al*. found that the low expression of *lncRNA-LET* is involved in cell invasion under hypoxic or normoxic conditions and its overexpression can inhibit the metastasis of HCC *in vivo* [47]. Additionally, a recent study revealed *lncRNA-ATB*, an lncRNA activated by TGF-β, can induce EMT and cell invasion *in vitro* and promote the invasion-metastasis cascade of HCC cells *in vivo* [48].

### 3.3. HCC Apoptosis

Some studies have demonstrated that lncRNAs are involved in HCC via acting on cell apoptosis. Braconi *et al*. revealed that *MEG3* expression was markedly reduced in four human HCC cell lines, compared with normal hepatocytes, and enforced expression of *MEG3* in HCC cells significantly decreased both anchorage-dependent and -independent cell growth, and induced apoptosis [42]. Yang *et al*. found that the TSA-induced *uc002mbe.2* expression was positively correlated with the apoptotic effect of TSA in HCC cells and knockdown the expression of *uc002mbe.2* significantly reduced TSA-induced apoptosis of Huh7 cells [50]. In addition, Xu *et al*. demonstrated that knockdown of the expression of *URHC* could promote apoptosis of HCC cells [51].

## 4. Molecular Mechanisms of LncRNAs in Hepatocellular Carcinoma

### 4.1. LncRNA-Protein Interaction

**Figure 1:**
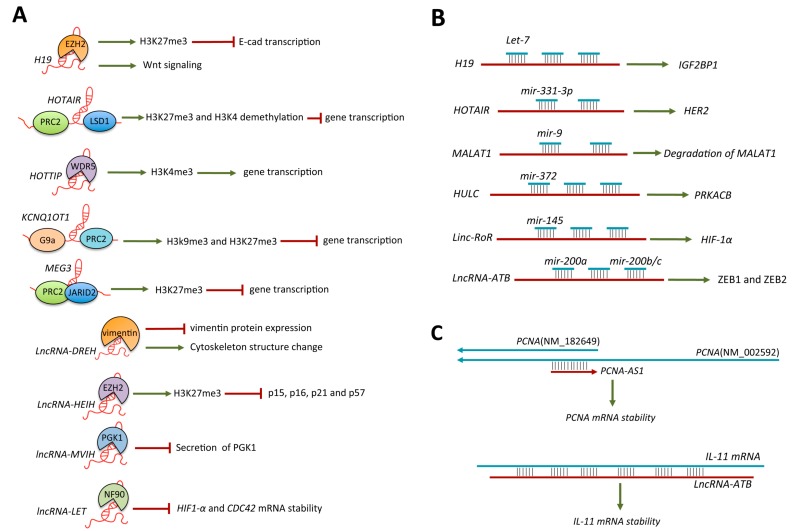
Overview of the molecular mechanisms of lncRNAs in HCC. (**A**) LncRNA-protein interaction; (**B**) LncRNA–microRNA interaction; and (**C**) LncRNA–mRNA interaction.

A large number of studies have revealed that many lncRNAs exert their function through interaction with proteins or protein complexes, especially with epigenetic complexes, such as polycomb repressive complex 1 (PRC1) and polycomb repressive complex 2 (PRC2) [59]. Some HCC-related lncRNA have been demonstrated to play roles in tumorigenesis via forming ribonucleoprotein (RNP) (Figure 1A). For example, it has been found that *H19* can specifically associate with enhancer of zeste homolog 2 (EZH2), a key subunit of the PRC2 complex, and inhibit E-cad expression by directly suppressing E-cad transcription and by indirectly activating Wnt signaling [21]. Tsai *et al*. demonstrated that *HOTAIR* served as a scaffold for two distinct histone modification complexes, PRC2 and LSD1/CoREST/REST complex. The ability to tether two distinct complexes enables RNA-mediated assembly of PRC2 and LSD1 and coordinates targeting of PRC2 and LSD1 to chromatin for coupled H3K27 methylation and H3K4 demethylation [53]. Wang *et al*. revealed *HOTTIP* RNA could bind the adaptor protein WDR5 directly and targets WDR5/MLL complexes across HOXA, driving H3K4 trimethylation and gene transcription [29]. Pandey *et al*. found that *KCNQ1OT1* could interact with chromatin and with the H3K9- and H3K27-specific histone methyltransferases G9a and the PRC2 complex in a lineage-specific manner [54]. Kaneko *et al*. uncovered *MEG3* interacted with PRC2 mainly through the RBR of JARID2 and *MEG3* acts in *trans* on PRC2 and JARID2 by facilitating their recruitment to a subset of target genes [57]. It was found that *LncRNA-DREH* could specifically associate with protein vimentin, a type III intermediate filament (IF) and the major cytoskeletal component of mesenchymal cells [44]. A recent study found *LncRNA-HEIH* also can associate with EZH2, and this association is required for the repression of EZH2 target genes in HCC, involving *p15*, *p16*, *p21* and *p57* [45]. Yuan *et al*. demonstrated that *lncRNA-MVIH* could activate angiogenesis by interacting with PGK1, a protein secreted by tumor cells and inhibit angiogenesis, and inhibiting its secretion [46]. In addition, another study revealed that *lncRNA-LET* could bind to NF90, a double-stranded RNA-binding protein that has been implicated in the stabilization, transport, and translational control of many target mRNAs, and decreases *HIF1-α* and *CDC42* mRNA stability through its association with NF90 under hypoxic and normoxic conditions, respectively [47].

### 4.2. LncRNA-MicroRNA Interaction

Interestingly, several recent reports have provided a model that suggests that lncRNA may function as competing endogenous RNA (ceRNA) in modulating the concentration and biological functions of microRNAs. These lncRNAs act as miRNA “sponges” generally share microRNA response elements (MREs) with the transcripts of several important genes and inhibiting normal miRNA targeting activity on mRNA. Several HCC-related lncRNA have been identified as miRNA “sponges” (Figure 1B). For example, it is found that vertebrate *H19* harbors both canonical and non-canonical binding sites for the *let-7* family of microRNAs, which plays key roles in development and cancer. Kallen *et al*. demonstrate that *H19* modulates *let-7* availability by acting as a molecular sponge using *H19* knockdown and overexpression, as well as *in vivo* crosslinking and genome-wide transcriptome analysis [18]. Liu *et al*. revealed that *HOTAIR* could function as a competing endogenous RNA to regulate HER2 expression by sponging *miR-331-3p* in gastric cancer [52]. Leucci *et al*. demonstrated that *miR-9* could directly bind *MALAT1* RNA *in vivo* and regulate the *MALAT1* in the nucleus in an AGO2-dependent manner [55]. Han *et al*. found *miR-125b* could directly bind *MALAT1* and down-regulated the *MALAT1* in bladder cancer [56]. Wang *et al.* demonstrated that the *lncRNA-HULC* acts as ceRNA of the protein coding gene PRKACB and induces its increased translation by controlling the expression and activity of *miR-372* in HCC. Takahashi *et al*. revealed that *linc-RoR* functioned as miRNA sponge to limit endogenous *miR-145* that can modulate the expression of key effectors of the hypoxia response such as *HIF-1α* expression in HCC. In addition, Yuan *et al*. found that *lncRNA-ATB* up-regulated ZEB1 and ZEB2 by competitively binding the *miR-200* family and then induced EMT and invasion of HCC [48]. However, it is noteworthy that a recent study from Bartel and Stoffel lab found target derepression of ceRNAs was in a threshold-like manner at high target site abundance and this threshold was insensitive to the effective levels of the miRNA via quantitating miRNA and target abundance. Strikingly, they concluded that modulation of miRNA target abundance is unlikely to cause signiﬁcant effects on gene expression and metabolism through a ceRNA effect *in vivo*, supporting that endogenous lncRNAs might actually function as ceRNA is highly unlikely [60].

### 4.3. LncRNA-mRNA Interaction

Accumulating evidence indicates that lncRNA can influence mRNA processing and post-transcriptional regulation via forming lncRNA-mRNA duplex depends on complementary base pairing, involving control of splicing, translation, and mRNA stability [61]. Some HCC-related lncRNA have been demonstrated that they can directly bind to target mRNA to exert post-transcriptional regulation (Figure 1C). For example, *PCNA-AS1*, antisense to PCNA, could increase *PCNA* mRNA stability via forming lncRNA–mRNA hybridization in HCC [43]. Additionally, Yuan *et al*. found that *lncRNA-ATB* specially increased the stability of *IL-11* mRNA, which depends on the binding of *IL-11* mRNA in HCC [48].

## 5. Conclusions and Perspectives

Hepatocellular carcinoma is a complex disease with multiple underlying pathogenic mechanisms caused by a variety of risk factors, and a better understanding of molecular mechanisms will help to identify potential molecular targets for diagnosis and therapy. In recent years, long noncoding RNA are gaining the attention of researchers in many fields, particularly in cancer and a large number of lncRNAs have been identified and there is an exponential growth of studies on the biological functions of lncRNAs in human cancers, including HCC.

LncRNAs often exhibit spatially and temporally-regulated expression patterns that that are expressed from specific tissue/cell types [62]. Their specificity makes them accurate biomarkers for cancer diagnostics. Furthermore, it has been demonstrated that cancer-specific lncRNAs can be detectable in plasma and urine of patients [63,64,65]. For example, *HULC*, an lncRNA highly up-regulated in liver cancer and positively related with Edmondson histological grades or with hepatitis B (HBV)-positive status, could be detected in the plasma of HCC patients compared to healthy controls and its higher detection rates were observed in the plasma of patients with higher Edmondson grades or with HBV-positive status [63]. The novel potential biomarkers can be discovered through certain types of highly expressed cancer-associated lncRNAs.

Therapeutic benefit can be obtained through RNA-based therapeutic strategies, such as siRNA and microRNA, or using small molecule compounds designed specifically to interact with target lncRNAs or ribonucleoprotein complexes. The nucleotides drugs can be effectively delivered to the liver using viral and non-viral systems. For viral mediated delivery, several types of viral vectors can be used, such as adenoviral and retroviral vector. Viral vector-mediated RNA delivery to liver can be achieved via the hepatic artery, portal vein, or bile duct or by direct injection to the liver [66]. For non-viral approaches, a suite of synthetic delivery carriers for liver targeting has been developed, such as galactosylated liposomes [67], poly-l-glutamic acid-coated liposomes [68], octaarginine (R8)-modified lipid nanoparticles [69], pH-triggered and PEGylated nanoparticles [70].

Although the roles played by lncRNAs in HCC have just begun to be revealed, with rapid development of high throughput detection technologies, such as microarray and RNA-sequencing and available bioinformatics tools for lncRNAs functional analysis, an increasing number of HCC-related lncRNAs will be identified and characterized. This will provide new insights into the complicated lncRNAs regulatory network, and ultimately provide novel strategies for HCC clinical diagnosis and treatment.
